# Characterizing and measuring bias in sequence data

**DOI:** 10.1186/gb-2013-14-5-r51

**Published:** 2013-05-29

**Authors:** Michael G Ross, Carsten Russ, Maura Costello, Andrew Hollinger, Niall J Lennon, Ryan Hegarty, Chad Nusbaum, David B Jaffe

**Affiliations:** 1 The Broad Institute, 7 Cambridge Center, Cambridge, MA 02142, USA

## Abstract

**Background:**

DNA sequencing technologies deviate from the ideal uniform distribution of reads. These biases impair scientific and medical applications. Accordingly, we have developed computational methods for discovering, describing and measuring bias.

**Results:**

We applied these methods to the Illumina, Ion Torrent, Pacific Biosciences and Complete Genomics sequencing platforms, using data from human and from a set of microbes with diverse base compositions. As in previous work, library construction conditions significantly influence sequencing bias. Pacific Biosciences coverage levels are the least biased, followed by Illumina, although all technologies exhibit error-rate biases in high- and low-GC regions and at long homopolymer runs. The GC-rich regions prone to low coverage include a number of human promoters, so we therefore catalog 1,000 that were exceptionally resistant to sequencing. Our results indicate that combining data from two technologies can reduce coverage bias if the biases in the component technologies are complementary and of similar magnitude. Analysis of Illumina data representing 120-fold coverage of a well-studied human sample reveals that 0.20% of the autosomal genome was covered at less than 10% of the genome-wide average. Excluding locations that were similar to known bias motifs or likely due to sample-reference variations left only 0.045% of the autosomal genome with unexplained poor coverage.

**Conclusions:**

The assays presented in this paper provide a comprehensive view of sequencing bias, which can be used to drive laboratory improvements and to monitor production processes. Development guided by these assays should result in improved genome assemblies and better coverage of biologically important loci.

## Background

**Figure 1:**
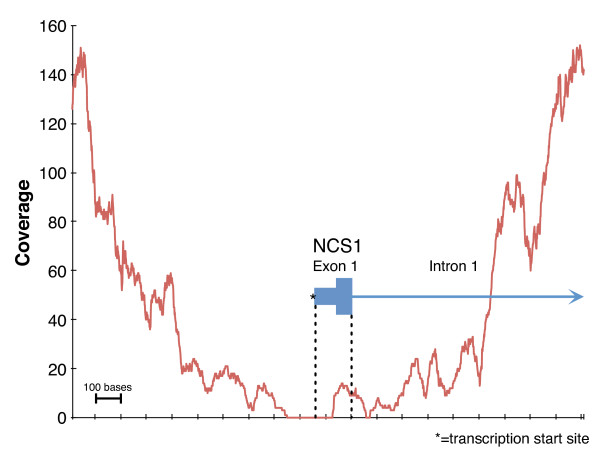
**Diagram illustrating the low coverage of *NCS1 *exon 1 in 198× Illumina HiSeq shotgun data**. The first 72 bases of the first exon of human gene *NCS1*, including the transcription start site, were uncovered in a 198× whole-genome shotgun data set (#A2). The displayed 2,000 base region is chromosome 9:132,933,910-132,935,910. *NCS1 *encodes calcium-binding proteins that regulate neurotransmitter release [1].

Ideal whole-genome shotgun DNA sequencing would distribute reads uniformly across the genome and without sequence-dependent variations in quality. All existing sequencing technologies fall short of this ideal and exhibit various types and degrees of bias. Sequencing bias degrades genomic data applications, including genome assembly and variation discovery, which rely on genome-wide coverage. Undercovered regions might lead to a missed SNP in an important region or cause an assembler to produce shorter contigs. For example, Figure 1 plots the coverage of the transcription start site and first exon of human gene *NCS1*, which encodes a neurotransmitter regulator [1], in whole-genome shotgun sequencing (data set A2). Despite 198-fold mean coverage of the genome, the first 72 bases of this exon are completely uncovered. This type of bias can reduce the effectiveness of biological and medical research. Recently published work on drug-resistant tuberculosis identified thousands of zero-coverage sites in an entire class of the bacterium's genes, despite sequencing to an average depth of 134× [2]. Alleviating gaps or dips in coverage through additional reads inflates sequencing costs, and may have limited effectiveness. For these reasons, improving our knowledge of sequencing bias is essential to improving the utility of DNA sequencing data.

Our goal in this work was to develop a rigorous method for discovering and monitoring coverage and error biases, then to apply it to data from a wide range of sequencing platforms (Illumina HiSeq and MiSeq, Ion Torrent PGM, Pacific Biosciences RS, and the Complete Genomics sequencing service). This study complements previous work in the field [3-7].

Bias manifests in multiple ways. Coverage bias is a deviation from the uniform distribution of reads across the genome. Similarly, error bias is a deviation from the expectation of uniform mismatch, insertion, and deletion rates in reads across the genome. This paper focuses primarily on coverage bias because it is the most damaging sequencing failure.

Sequencing technologies are vulnerable to multiple sources of bias. Methods based on bacterial cloning and Sanger-chemistry sequencing [8] were subject to many coverage-reducing biases, notably at GC extremes, palindromes, inverted repeats, and sequences toxic to the bacterial host [9-17]. Illumina sequencing [18] has been shown to lose coverage in regions of high or low GC [19-22], a phenomenon also seen in other 'next-generation' technologies [3,6]. PCR amplification during library construction is a known source of undercoverage of GC-extreme regions [20,21] and similar biases may also be introduced during bridge PCR for cluster amplification on the Illumina flowcell [23]. Illumina strand-specific errors can lead to coverage biases by impairing aligner performance [24]. Ion Torrent [25], like 454 [26], utilizes a terminator-free chemistry that may limit its ability to accurately sequence long homopolymers [4,27,28], and may also be sensitive to coverage biases introduced by emulsion PCR in library construction. Complete Genomics [29] also uses amplification along with a complex library construction process. The Pacific Biosciences [30] process is amplification-free; therefore, one might expect it to exhibit lower levels of coverage bias than the other technologies.

In addition to sources in the wet lab, bias can be introduced by any of the computational steps in the sequencing pipeline. Signal-processing and base calling limitations could result in under-representation or increased error rates in some locations, as can inaccurate alignment. An inaccurate reference or sample-reference differences can cause coverage or accuracy variations that may be misdiagnosed as sequencing bias. Therefore, detecting bias is only the first step and must be followed by more detailed experiments to assign responsibility to the library preparation, sequencing, or computational stages.

We employ two methodologies for measuring bias. Per-base bias measurements, which rely on deep-coverage sequencing, are hypothesis-free and ideal for discovering new types of bias. Motif bias measurements, which require only shallow-coverage sequencing, are ideal for comparisons across experimental conditions and for monitoring ongoing sequencing pipeline performance at known bias-prone sequence contexts and locations. Bias motif monitoring plays a useful role by providing a critical metric in determining and ameliorating the sources of sequencing bias. Together these methodologies can be used to compare platforms, to measure the utility of combining data from multiple platforms, and to determine the extent to which coverage bias is described by the statistics of known motifs.

## Results and discussion

### Per-base bias

We begin by defining the bias statistics. The fundamental statistic of coverage bias is 'relative coverage', which is defined as:

$$\frac{\text{coverage}\;\text{of}\;\text{a}\;\text{gieven}\;\text{reference}\;\text{base}\;\text{in}\;\text{a}\;\text{genome}}{\text{mean}\;\text{coverage}\;\text{of}\;\text{all}\;\text{reference}\;\text{bases}}.$$

The coverage of a given reference base is computed by counting the number of read bases mapped to it in an alignment (see Materials and methods). The mean coverage is computed by averaging this value across every base in the reference. Then the relative coverage for a particular base is computed as the ratio of these values. A relative coverage of 1 indicates that a particular base is covered at the expected average rate. A relative coverage above 1 indicates higher than expected coverage and below 1 indicates lower than expected coverage.

Some reads cannot be mapped to a single locus, and the probability of ambiguous mapping increases as reads become shorter or less accurate. Ambiguous mapping is also more likely for reads that derive from repetitive or low complexity regions of the genome, including some regions with extreme GC content. To solve this problem, we rely on the aligner employing a policy of random assignment when there are multiple 'best' alignments. This provides the optimal measurement of coverage bias given the data: it is impossible to know whether specific locations are evenly represented, but we can nonetheless expect to accurately assess the coverage of classes of bases as defined by some local sequence context (for example, involving GC content, and so on). All the alignment algorithms used in this work (see Materials and methods) use this random-placement policy.

Bases having low relative coverage are of particular interest, provided that the low coverage is not an accident of sample size. For example, at 20-fold mean coverage, some bases whose 'true' relative coverage is 1 (corresponding to an expectation of 20 overlapping reads), will occasionally have measured relative coverage of 0.5 (corresponding to an observation of 10 overlapping reads), as that measurement is only off by $\left(20-10\right)/\sqrt{20}\approx 2.2$ standard deviations (based on a Poisson model). Thus, deep sequencing is required to accurately identify bases having low relative coverage.

### Motif bias

Typically, only a small fraction of a genome has 'low' relative coverage. For example, 198-fold mean coverage of the human genome by Illumina HiSeq 2000 version 2 chemistry only left 0.23% of bases undercovered by a factor of 10 or more (data set A2). At first glance, this portion of the genome appears minuscule, but if the data were unbiased, we would expect no bases to have such a low level of coverage (more than 12 standard deviations less than the mean). Additionally, this small undercovered fraction included important loci. For example, this deep-coverage HiSeq data set contained no reads overlapping the transcription start sites of several genes associated with early development, transcriptional regulation, cell-cell adhesion, actin binding, neural development, and intracellular signaling (for an example, see Figure 1). Thus, understanding the specific nature of undercovered sequences is important. We approached this problem in two ways: by evaluating specific biologically important regions of the genome that are significantly undercovered, and by identifying specific sequence motifs that are systematically undercovered. Anecdotal results suggested that many transcription start sites or first exons in the human genome tend to have poor coverage. By a systematic analysis of these regions we defined the 1,000 with the lowest relative coverage based on low coverage by an Illumina data set, which we term the 'bad promoters' list (see Materials and methods). The bad promoters are, like many exons, GC-rich (averaging 79% GC composition).

It is well established that extreme base composition is associated with bias in multiple technologies [3,4,6,13,14,19-22,27]. In this work, we define specific base composition categories that are associated with bias, which we refer to as 'motifs'. Motif bias statistics can be measured accurately with much less data than per-base statistics (see below). They are also valuable because they can suggest underlying causes of bias that can then be investigated in laboratory experiments and can be used to track performance of attempted process improvements.

We developed a list of five bias motifs that encapsulate several common sources of coverage bias:

• GC ≤ 10%, 200-base regions in which the middle 100 bases have ≤10% GC content;

• GC ≥ 75%, 200-base regions in which the middle 100 bases have ≥75% GC content;

• GC ≥ 85%, 200-base regions in which the middle 100 bases have ≥85% GC content;

• (AT)^15^, 130-base regions in which the middle 30 bases are repeated AT dinucleotides;

• G|C ≥ 80%, 130-base regions in which the middle 30 bases are either 80% Gs or 80% Cs (and, therefore, match long G or C homopolymers).

For human data, we added a sixth motif based on the aforementioned list of undercovered transcription start sites: the 1,000 empirically defined 'bad promoter' 200-base intervals from the human genome (as defined above; coordinates reported in Additional file 1).

The 'special' motifs (AT)^15 ^and G|C ≥ 80% are included based on anecdotal evidence that contig breaks in assemblies are frequently associated with these motifs. The extents of all the motifs in the reference genomes studied in this paper are presented in Table 1. The decision to attend to regions of 100 to 200 bases was an empirical choice influenced by considerations such as the distribution of fragment sizes in our Illumina libraries. Computing our statistics using larger or smaller regions might make different biases apparent depending on the properties of the assayed data set.

**Table 1:** Genomes and motifs

		GC extremes			Special motifs		
			
Sample	Genome size	GC ≤ 10%	GC ≥ 75%	GC ≥ 85%	(AT)^15^	G|C ≥ 80%	Bad promoters
*P. falciparum*	23,263,391	10,030,724 (43%)	0	0	1,258,098 (5.4%)	0	-
*E. coli*	4,638,920	0	2,705 (0.058%)	0	0	0	-
*R. sphaeroides*	4,131,450	0	2,479,536 (60%)	90,207 (2.2%)	0	0	-
Human	2,684,573,005	6,228,029 (0.23%)	20,669,681 (0.77%)	2,980,450 (0.11%)	1,253,245 (0.047%)	802,554 (0.030%)	190,041 (0.0071%)

For each genome sequenced as part of this work, we show its size in bases, along with the number of bases of each bias motif (see text). Only unambiguous (A, C, T, or G) bases from each reference are included. Plasmids, mitochondria, and sex chromosomes were excluded from the counts.

**Figure 2:**
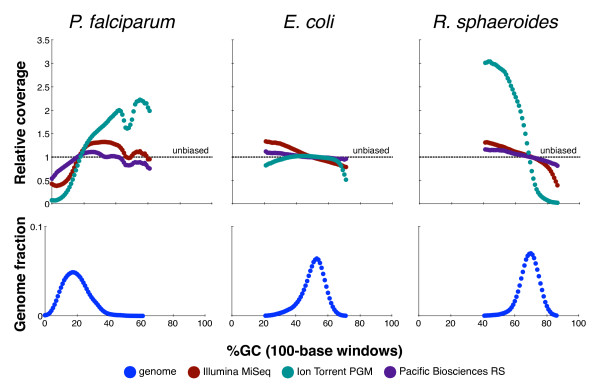
**GC-bias plots for three microbial genomes**. Top: plots showing the relative coverage GC-bias for Illumina MiSeq, Ion Torrent PGM, and Pacific Biosciences RS on the *P. falciparum *(19% GC), *E. coli *(51%), and *R. sphaeroides *(69%) genomes (Table 2, data sets 1 to 9). Unbiased coverage would be represented by a horizontal line at a relative coverage = 1 (black dashed line). Relative coverage is only plotted for GC percentages for which there are at least 1,000 100-base windows in the genome. Bottom: the GC composition distribution of each genome.

These motifs focus on known trouble spots. Because GC base composition is frequently implicated in coverage bias, it is also useful to measure the relative coverage across the entire GC spectrum by grouping all 100-base sliding windows across the genome by their GC content and reporting the average relative coverage for each GC-content percentage (in effect defining a motif for each percentage). The results can be presented as a GC-bias plot, as exemplified in Figure 2. Unbiased sequencing would be unaffected by GC composition, resulting in a flat line along relative coverage = 1.

Because motifs are typically represented by many loci in a genome, the number of reads incident upon a motif is much larger than the number of reads incident upon a single base, and hence the relative coverage of a motif (that is, the mean of the relative coverages of its constituent bases) can be accurately measured even with low sequencing coverage.

Table 2 presents relative coverage of the six motifs across 16 data sets. From data set 14 (120-fold coverage of Illumina from the human genome) we also chose ten 0.5-fold random subsets, in each case computing the relative coverage across the motifs. For each motif, we show (data set 14') the mean of these ten measurements, which for all motifs were within 0.01 of the full sample value, and the observed standard deviation, which for all motifs was approximately 0.02 or less. This shows that for the human genome, relative coverage of the six motifs can be accurately assayed using low coverage.

**Table 2:** Data sets and their relative coverage on bias motifs

					Relative coverage					
					
Data set					GC extremes			Special motifs		
		
Sample	#	Library method	Sequencing platform	Coverage (x)	GC ≤ 10%	GC ≥ 75%	GC ≥ 85%	(AT)^15^	G|C ≥ 80%	Bad promoters
*P. falciparum*	1	Fisher *et al*.^a^ with Kapa reagents	Illumina MiSeq	150	0.58	-	-	0.43	-	-
3D7	2	Ion Torrent standard	Ion Torrent PGM	103	0.39	-	-	0.11	-	-
	3	Pacific Biosciences standard	Pacific Biosciences RS	104	0.89	-	-	0.85	-	-
*E. coli*	4	Fisher *et al*.^a^ with Kapa reagents	Illumina MiSeq	380	-	0.82	-	-	-	-
K12 MG1655	5	Ion Torrent standard	Ion Torrent PGM	311	-	0.31	-	-	-	-
	6	Pacific Biosciences standard	Pacific Biosciences RS	115	-	0.97	-	-	-	-
*R. sphaeroides*	7	Fisher *et al*.^a^ with Kapa reagents	Illumina MiSeq	388	-	0.94	0.60	-	-	-
2.4.1	8	Ion Torrent standard	Ion Torrent PGM	302	-	0.39	0.10	-	-	-
	9	Pacific Biosciences standard	Pacific Biosciences RS	142	-	0.97	0.87	-	-	-
Human	10	Aird *et al*. with Phusion	Illumina HiSeq v2	028	0.58	0.27	0.071	0.38	0.19	0.027
NA12878	11	Aird *et al*. with Phusion+betaine	Illumina HiSeq v2	048	0.44	0.44	0.28	0.26	0.20	0.14
	12	Aird *et al*. with AccuPrime	Illumina HiSeq v2	075	0.42	0.42	0.23	0.23	0.38	0.16
	13	Fisher *et al*.^a^	Illumina HiSeq v3	070	0.29	1.1	0.56	0.23	0.44	0.39
	14	Fisher *et al*.^a^ with Kapa reagents	Illumina HiSeq v3	120	0.41	0.88	0.48	0.25	0.65	0.36
	14'	Fisher *et al*.^a^ with Kapa reagents	Illumina HiSeq v3	000.5	0.41 ± 0.0032	0.88 ± 0.0047	0.48 ± 0.0067	0.25 ± 0.0042	0.65 ± 0.012	0.37 ± 0.022
	15	Ion Torrent standard	Ion Torrent PGM	001.1	0.27	0.36	0.068	0.19	0.26	0.046
	16	Complete Genomics standard	Complete Genomics	079	0.24	0.53	0.18	0.28	0.61	0.092

^a^Low-input variation of Fisher *et al*. [31] (see Materials and methods). Data sets from samples, library construction methods and sequencing platforms are shown, along with their total coverage of the genome, and relative coverage, for each of five bias motifs and a set of 'bad promoters' (see text). Entries are blank if the samples' genome had no instances of the given motif. Data set 14' is the summary of ten random subsamplings from data set 14, with coverage reduced to 0.5×, and we show the mean and standard deviations for the relative coverage measurements from it (see text).

### Comparing bias across technologies

#### Bias in a GC-spanning set of microbes

To assess the bias profile across technologies efficiently, we generated data from three microbial genomes that together span a wide range of GC base composition: *Plasmodium falciparum *(mean 19% GC), *Escherichia coli *(51%) and *Rhodobacter sphaeroides *(69%). All three genomes have finished reference sequences, thus facilitating a definitive analysis (see Materials and methods). Only data from Illumina (MiSeq), Ion Torrent and Pacific Biosciences were examined, not from Complete Genomics, which generates only human sequencing data. For all analyses, we note that although results are categorized by sequencing technology, in fact bias can also be introduced by library construction, and that disentanglement of these variables would require additional experiments. For the following bacterial genome analysis, Illumina libraries were made following a low-input variation of the protocol detailed in Fisher *et al. *[31], modified with Kapa Biosystems reagents (see Materials and methods), and both Ion Torrent and Pacific Bioscience libraries were generated using the respective manufacturers' reagents and recommended protocols (see Materials and methods).

We first asked how much of each of the three genomes was undercovered by each of the three technologies (Table 3, 1 to 9, italics), ensuring comparability by downsampling each data set to 100-fold coverage, and testing several levels of relative coverage (0.5, 0.25, 0.1 and no coverage). While modest variation was seen for *E. coli *on all three platforms, the results for the GC-extreme genomes were striking. For example, the fraction of the GC-poor *P. falciparum *genome that had relative coverage ≤0.25 (that is, four-fold undercovered or worse) ranged from 0.33% in Pacific Biosciences data (best) to 3.7% in Illumina data to 22% in Ion Torrent data (worst). In the GC-rich *R. sphaeroides *genome, the four-fold undercoverage fractions were 0.0071% for Pacific Biosciences (best), 0.39% for Illumina, and 36% for Ion Torrent (worst). The better performance of Pacific Biosciences is probably attributable to the lack of any amplification in their process (compare [20,21]).

**Table 3:** Percentage of undercovered microbial genome given 100× coverage

Data set			Relative coverage thresholds (% of genome)			
	
Sample	#	Sequencing platform	= 0	≤0.1	≤0.25	≤0.5
*P. falciparum *3D7	*1*	*Illumina MiSeq*	0.010	0.18	3.7	24
	*2*	*Ion Torrent PGM*	2.6	14	22	33
	*3*	*Pacific Riosciences RS*	0.012	0.13	0.33	2.7
	1+2	Illumina + Ion Torrent	0.0096	1.1	12	30
	2+3	Ion Torrent + Pac Bio	0.0062	0.097	1.6	17
	3+1	Pac Bio + Illumina	0.0051	0.040	0.33	7.9
*E. coli *K12 MG1655	*4*	*Illumina MiSeq*	0.00022	0.0019	0.019	0.54
	*5*	*Ion Torrent PGM*	0.00047	0.013	0.046	0.27
	*6*	*Pacific Biosciences RS*	0	0.00075	0.030	0.36
	4+5	Illumina + Ion Torrent	0	0.0012	0.0053	0.075
	5+6	Ion Torrent + Pac Bio	0	0.00037	0.0018	0.054
	6+4	Pac Bio + Illumina	0	0.00026	0.0012	0.061
*R. sphaeroides *2.4.1	*7*	*Illumina MiSeq*	0.00094	0.045	0.39	2.7
	*8*	*Ion Torrent PGM*	0.88	19	36	47
	*9*	*Pacific Biosciences RS*	0.000048	0.0021	0.0071	0.067
	7+8	Illumina + Ion Torrent	0.0038	0.23	1.8	19
	8+9	Ion Torrent + Pac Bio	0.000024	0.00058	0.14	16
	9+7	Pac Bio + Illumina	0.00012	0.0018	0.017	0.49

The percentage of each microbial genome left completely uncovered as well as ten-fold, four-fold, and two-fold undercovered given 100× (randomly downsampled) coverage of Illumina MiSeq, Ion Torrent PGM, and Pacific Biosciences RS data (data sets in italics; Table 2). Also included are the same statistics from data sets that combine 50× coverage from each pair of sequencing technologies.

Next, to better understand what parts of the genome were undercovered, we generated GC-bias plots (Figure 2), showing relative coverage at each GC level (and for context, the fraction of the genome at each level). These plots provide fine detail but also mirror the preceding conclusions, exhibiting the same hierarchy at GC extremes. For example, Ion Torrent coverage dropped severely below 10% and above 75% GC. On the other hand, all three technologies provided nearly even coverage of the moderate-GC range (30 to 70%) in *E. coli*. At the lowest GC, even Pacific Biosciences showed approximately two-fold coverage reduction, perhaps attributable to dissociation of fragment ends in adapter ligation, a phenomenon that could apply to all three technologies.

Finally, Table 2 (data sets 1 to 9) presents the relative coverage of the previously described motifs, although not all are present in each sample (the G|C ≥ 80% motif is absent in all of the microbes, and the set of bad promoters was only defined for the human genome). We note that the single statistic of relative coverage for the GC ≥ 85% motif provided a suitable assay for bias on *R. sphaeroides*, with Pacific Biosciences scoring 0.87 (best), Illumina 0.60 and Ion Torrent 0.10 (worst), while GC ≥ 75% did not clearly distinguish between Illumina and Pacific Biosciences data. The GC ≤ 10% motif was similarly useful for *P. falciparum*, with Pacific Biosciences scoring 0.89 (best), Illumina 0.58, and Ion Torrent 0.39 (worst). For these data, the (AT)^15 ^motif also stood out, with Pacific Biosciences at 0.85, Illumina at 0.43, and Ion Torrent at 0.11. Importantly, just these few statistics provided a meaningful readout on the performance of the different technologies.

#### Bias on human samples

The human genome is far larger and more complex than the previously analyzed microbes and contains many examples of all of the motifs, as well as the 1,000 bad promoters (Table 1). We generated slightly more than one-fold coverage on the Ion Torrent PGM platform and 120-fold coverage on Illumina HiSeq. We also analyzed a 79-fold coverage data set generated by Complete Genomics. Complete Genomics sequencing, like Illumina and Ion Torrent, uses amplification in its process. We did not analyze the performance of Pacific Biosciences on human samples because, at the time of these experiments, the system's throughput made it impractical to generate sufficient coverage. To maximize comparability and avoid misinterpreting biological variation as sequencing variation, all data sets utilized the well-studied NA12878 sample [32] and were aligned to the Human Genome Assembly 19 (GRCh37) reference.

**Figure 3:**
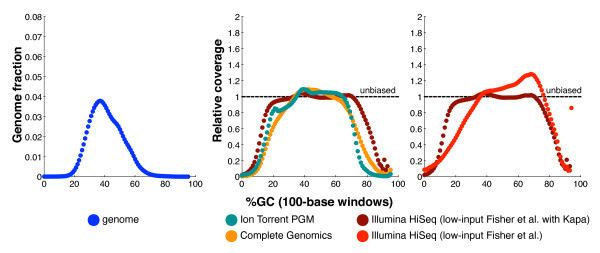
**GC-bias plots for the human genome**. Left: the GC composition distribution of the human genome (HG19, GRCh37). Center and right: GC-bias plots for several data sets from human NA12878. Unbiased coverage would be represented by a horizontal line at relative coverage = 1. Center: HiSeq v3 with sample-preparation reagents from Kapa Biosystems (Table 2, data set 14), Ion Torrent PGM (data set 15), and Complete Genomics data (data set 16). Right: HiSeq v3 with sample-preparation reagents from Kapa Biosystems (data set 14, as in center panel) and HiSeq v3 with the standard Fisher *et al. *[31] reagents (data set 13). Note that Illumina relative coverage exceeded the y-axis above 93% GC content. Relative coverage is only plotted for GC percentages for which there are at least 1,000 100-base windows in the genome.

Table 2 and Figure 3 show the motif results and bias curves comparing Illumina HiSeq (data set 14), Ion Torrent PGM (data set 15), and Complete Genomics (data set 16) coverage of NA12878. The HiSeq libraries were prepared using the low-input Fisher *et al*. protocol [31] modified with Kapa Biosystems reagents (see Materials and methods), the other libraries used the manufacturers' standard protocols (see Materials and methods). We use data set 14 to represent HiSeq performance, rather than the other HiSeq human data sets in Table 2, because it represents our current best Illumina library construction protocol. Of the data sets tested, the bias curves clearly suggest that the Illumina HiSeq data provided the most even coverage of the human genome. Complete Genomics coverage dropped more severely at both GC extremes and only provided 0.092 relative coverage of the bad promoters, compared to 0.36 relative coverage by HiSeq. The Ion Torrent coverage dropped even more quickly than Complete Genomics as GC increased and only provided 0.046 relative coverage of the bad promoters. Ion Torrent also had the worst performance of these three data sets on the (AT)^15 ^and G|C ≥ 80% motifs.

In Table 2 we can also see how updates to the Illumina HiSeq platform have affected bias. Notably, the HiSeq version 3 data (data sets 13 and 14) show better coverage of high-GC motifs and the bad promoters compared to the HiSeq version 2 data (data sets 10 to 12). We have also compared the standard list of bad promoters, computed from HiSeq version 2 data, to a new list computed from HiSeq version 3 data (see Materials and methods and Additional files 1 and 2 for details). The lists have 47% of their bases in common, which indicates that many bad promoters are still resistant to sequencing despite Illumina's improvements.

The inter-platform GC-bias comparisons on human and microbial samples presented above are broadly compatible with previously published work [3,5]. However, we clearly observed more bias between 60% and 70% GC on *R. sphaeroides *in Ion Torrent data than on MiSeq data, while Liu *et al. *[7] found the reverse when comparing Ion Torrent to HiSeq. Our *R. sphaeroides *results are compatible with the results reported by Ion Torrent for the high-GC *Rhodopseudomonas palustris *genome [28].

### Comparing bias across libraries

Library construction methods affect evenness of coverage [20-22]. Table 2 includes human Illumina data produced using the methods described in Aird *et al. *[20] that are illustrative of this, showing a striking improvement at high GC when the PCR enzyme Phusion HF (data set 10) was supplemented by betaine (data set 11) or replaced by AccuPrime Taq HiFi (data set 12). Figure 3 shows a marked flattening of relative coverage between 15% and 70% GC when we replaced some reagents in the low-input Fisher *et al*. protocol (data set 13) [31] with reagents from Kapa Biosystems (data set 14) (see Materials and methods), although the large improvement at low-GC was partly offset by a small decline in high-GC coverage (Figure 3, Table 2). Oyola *et al. *[21] achieved a similar improvement in low-GC coverage of *P. falciparum *by utilizing Kapa HiFi enzymes and the PCR additive tetramethylammonium chloride in library construction.

It is also true that there can be variation in bias between 'technical replicates', data sets created from the same sample using the same protocols. For example, the HiSeq 'Kapa' human data set (data set 14) was created from three libraries and sequenced in fourteen lanes on two flowcells, with no deliberate variation in protocol at any point. Yet when bias statistics are computed lane-by-lane, one sees substantial variation in bias between libraries, and between flowcells - although not between lanes from the same library and flowcell (Table 4). Most notable is the between-flowcell variation of the G|C ≥ 80% motif, which is approximately two-fold undercovered in the first flowcell, but very well covered in the second. Possible sources of unexplained variation include variability of library construction instantiations, cluster amplification devices (cBot), flowcells, and HiSeq instruments that were used. Although variations between technical replicates are of interest, they are, for the most part, smaller than those observed between platforms.

**Table 4:** Per-lane bias statistics for Illumina HiSeq (Kapa) human NA12878

				Relative coverage					
				
Data set				GC extremes			Special motifs		
		
Flowcell	#	Lane	Library	GC ≤ 10%	GC ≥ 75%	GC ≥ 85%	(AT)^15^	G|C ≥ 80%	Bad promoters
C0G7VACXX	14a	1	A	0.39	0.93	0.49	0.25	0.53	0.37
	14b	2	A	0.39	0.93	0.50	0.25	0.53	0.39
	14c	3	B	0.41	0.86	0.46	0.25	0.50	0.36
	14d	5	B	0.41	0.85	0.45	0.26	0.50	0.36
	14e	6	C	0.42	0.83	0.38	0.26	0.49	0.30
	14f	7	C	0.42	0.83	0.37	0.26	0.49	0.31
D0K2WACXX	14g	1	B	0.41	0.89	0.55	0.25	0.85	0.38
	14h	2	B	0.40	0.89	0.56	0.25	0.85	0.39
	14i	3	B	0.41	0.89	0.56	0.25	0.86	0.40
	14j	4	A	0.39	0.96	0.61	0.25	0.96	0.41
	14k	5	C	0.42	0.85	0.43	0.26	0.70	0.32
	14l	6	C	0.43	0.85	0.43	0.26	0.68	0.33
	14m	7	C	0.42	0.86	0.44	0.26	0.71	0.32
	14n	8	A	0.39	0.97	0.62	0.25	0.95	0.41

Bias statistics for the lanes and libraries that compose the HiSeq v3 (low-input Fisher *et al*. [31] with Kapa reagents) human data set (data set 14 in Table 2). Letters identify the libraries (A = Solexa-77484, B = Solexa-77486, C = Solexa-77483), which were all made using the same protocol.

It is now possible to create 'PCR-free' Illumina libraries, in which there is no DNA amplification prior to cluster generation and sequencing. A comparison of libraries prepared with our standard Fisher *et al*. protocol and a PCR-free protocol (Table S1 in Additional file 3) reveals that the PCR-free libraries lead to less bias across all bias motifs on *P. falciparum*, *E. coli*, and *R. sphaeroides *samples. On human samples, PCR-free library construction produced improved coverage of all motifs except for GC ≥ 75% and G|C ≥ 80%. Additionally, the bad promoters, although improved, were still two-fold undercovered. These results suggest that PCR-free library construction reduces, but does not cure, coverage bias.

#### Coverage complementarity

Combining the outputs of multiple sequencing technologies might create a composite data set whose overall bias is reduced. Two technologies provide complementary coverage if, on the same sample, they tend to fill in each other's low-coverage regions. Complementary technology mixtures should have bias statistics that are better than either one of the components. Precedent for this approach stretches back to the practice of combining data from dye-terminator and dye-primer chemistries in Sanger sequencing to reduce error biases [33]. Note that there can be other benefits from mixing technologies, by taking advantage of a broader range of complementary properties (and not just bias). For example, for genome assembly there are benefits from combining the long, relatively unbiased but lower accuracy reads from Pacific Biosciences with shorter Illumina reads that provide per-base accuracy [34-36].

To evaluate complementarity, we created mixed-technology microbial data sets for each possible platform pairing (MiSeq and Ion Torrent, Ion Torrent and Pacific Biosciences, Pacific Biosciences and MiSeq) using the previously described data sets (data sets 1 to 9). Each pairing consisted of 100-fold total coverage, composed of 50-fold randomly sampled coverage from each component technology. Then we measured the fraction of each genome that fell beneath several relative coverage thresholds, comparing those results to the undercoverage values from 100-fold 'pure' coverage from the component technologies (Table 3). If the coverage biases were complementary, we would expect that the undercoverage fractions from the mixed data sets would be smaller than those measured in the component pure data sets. This did happen in some cases. For *E. coli*, using a mixture of Illumina and Ion Torrent data, the two-fold undercovered fraction was 0.075%, compared to 0.54% and 0.27%, respectively, for the two technologies taken separately. Similar improvements occurred for *E. coli *with other platform combinations. However, for the other organisms, for the technologies tested, combining data did not reduce the overall level of bias. In most cases, one technology had much lower bias than the other and mixing tended to result in an intermediate level of bias. Therefore, in these cases, mixing provided no coverage benefit; lower bias could have been achieved by only using data from the lower bias technology.

#### Error biases

**Figure 4:**
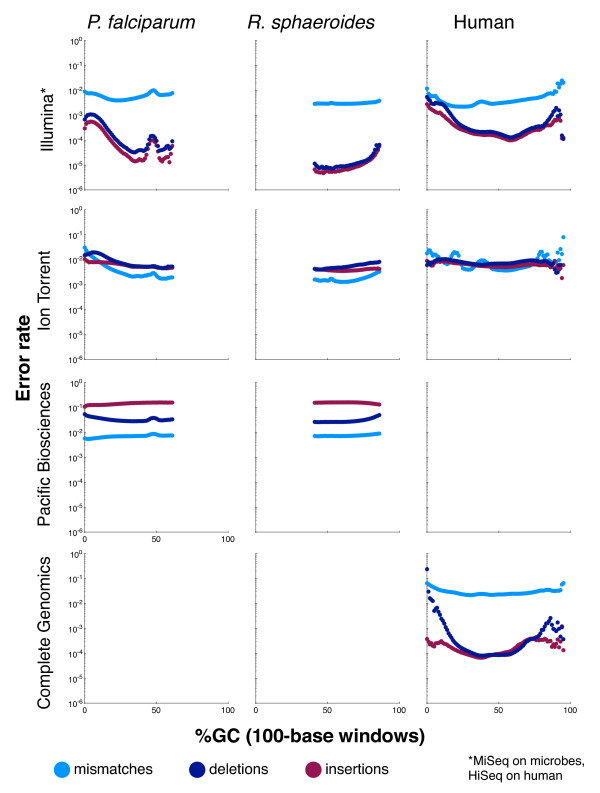
**Error rates as a function of GC composition**. Each graph shows mismatch (light blue), deletion (dark blue), and insertion (maroon) rates (y-axis) as a function of GC composition (x-axis). Data are shown for the Ion Torrent PGM from three organisms (*P. falciparum*, *R. sphaeroides*, and human), for the Illumina MiSeq on the two microbes, for the Illumina HiSeq on human, for Pacific Biosciences from the two microbes and from Complete Genomics for human (Table 2, data sets 1 to 3, 7 to 9, and 14 to 16). For human we note that *bona fide *differences between the sample and the reference sequence were recorded as errors. Error rates are only plotted for GC percentages for which there are at least 1,000 100-base windows in the genome.

**Figure 5:**
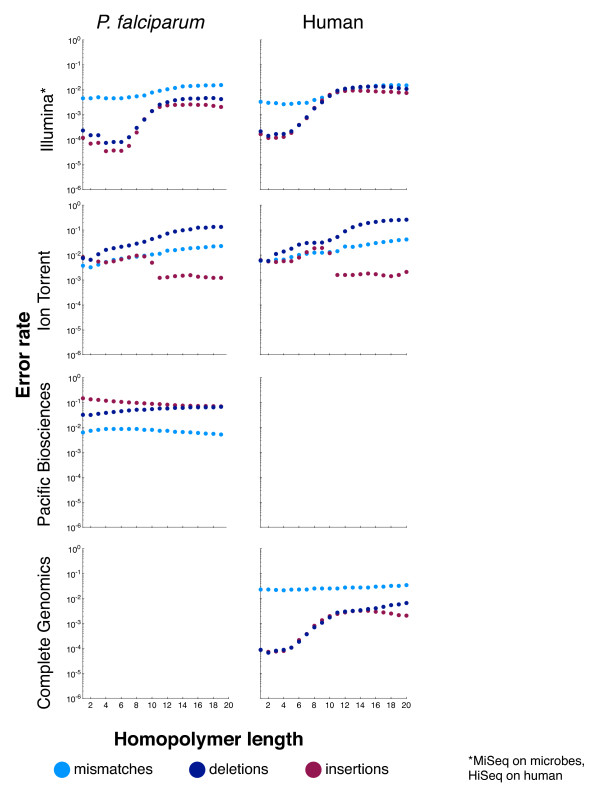
**Error rates as a function of homopolymer length**. Each graph shows mismatch (light blue), deletion (dark blue), and insertion (maroon) rates (y-axis) within homopolymers of various lengths (x-axis). Data are plotted from *P. falciparum *and human as available (Table 2, data sets 1 to 3 and 14 to 16). For human we note that *bona fide *differences between the sample and the reference sequence were recorded as errors.

While coverage bias is an important sequencing metric, it ignores possible variations in sequence accuracy. For many applications, decreases in accuracy could offset the advantages of better relative coverage in difficult regions. To compare between platforms and assess the influence of sequence context, Figure 4 plots the mismatch, deletion, and insertion rates on *P. falciparum*, *R. sphaeroides*, and human for the four surveyed technologies, as a function of GC content, whereas Figure 5 plots the same as a function of homopolymer length. A logarithmic scale is used to facilitate comparison between technologies and between error types because rates vary greatly. Table 5 lists the genome-wide error rates for the four platforms. For human, the reported errors include *bona fide *differences between the NA12878 sample and the reference sequence, and hence the error rates were somewhat inflated. When Illumina NA12878 data (data set 14) were aligned to an NA12878-specific reference [37], the mismatch rate declined by 40%, and the indel rate declined by 80% (Table S2 in Additional file 3). Because of their larger magnitude, a similar experiment yielded no substantial change in the Ion Torrent error rates.

**Table 5:** Sequencing technology error rates

Data set			Fractional error rate			
	
Sample	#	Platform	Mismatches	Deletions	Insertions	Total
*P. falciparum*	1	Illumina MiSeq	0.0046	0.00021	0.00011	0.0049
	2	Ion Torrent PGM	0.0038	0.0090	0.0068	0.020
	3	Pacific Biosciences RS	0.0068	0.033	0.14	0.18
*E. coli*	4	Illumina MiSeq	0.0036	0.0000097	0.0000051	0.0037
	5	Ion Torrent PGM	0.0018	0.0053	0.0044	0.012
	6	Pacific Biosciences RS	0.0077	0.032	0.17	0.21
*R. sphaeroides*	7	Illumina MiSeq	0.0030	0.000018	0.0000089	0.0030
	8	Ion Torrent PGM	0.0014	0.0055	0.0037	0.011
	9	Pacific Biosciences RS	0.0076	0.029	0.16	0.20
Human	14	Illumina HiSeq	0.0030	0.00023	0.00017	0.0034
	15	Ion Torrent PGM	0.0060	0.0069	0.0057	0.019
	16	Complete Genomics	0.023	0.000099	0.000091	0.024

For a subset of the data sets in Table 2, we show the fractional rates of mismatch, deletion, and insertion, computed relative to coverage, inferred by comparison to the reference sequences. For human we note that *bona fide *differences between the sample and reference sequence were recorded as errors.

Briefly, while the details depend on the technology, these plots document changes in error rates at GC extremes and on long homopolymers, for every technology. For example, Illumina, which had very low insertion and deletion error rates, had a substantial rise in insertions and deletion rates at both GC extremes. The Ion Torrent insertion and deletion rates were more consistent, albeit higher than Illumina's, across a range of GC contents, but the mismatch rate was elevated at low and high-GC regions. As another example, we note that for Pacific Biosciences, the deletion rate rose at high GC, while the insertion rate declined. This behavior appears to result from lower signal-to-noise ratios for the dyes attached to G and C bases in C1 chemistry (personal communication, Edwin Hauw, Pacific Biosciences, USA). Complete Genomics showed consistent (relatively high) mismatch and (relatively low) insertion rates across the GC spectrum, but the deletion rate rose substantially at the extremes. Within long homopolymers, the behavior of insertion and deletion errors would depend on whether a technology systematically over- or under-reports homopolymer length. For example, as homopolymer lengths increased, Ion Torrent showed an increased deletion rate, but the insertion rate stayed about the same. In contrast, the insertion and deletion rates of Illumina data increased in longer homopolymers, which is consistent with their behavior in GC-extreme regions. In the Illumina and Ion Torrent human data, these trends were unchanged when the data were realigned to a sample-specific reference [37] that accounted for known biological variations (Figure S1 in Additional file 4, Figure S2 in Additional file 5). Similarly consistent with GC behavior are the decrease in insertions and increase in deletions observed in Pacific Biosciences data in long homopolymers. In general, the sequence-context dependence of error rates varied considerably from technology to technology.

PCR amplification in library construction is a source of error in sequencing data [38-40]. In a matched comparison, we found that our production libraries had lower error rates than a PCR-free protocol on *E. coli *and human samples, and only a slight increase in error rate on *R. sphaeroides *and *P. falciparum *(Table S3 in Additional file 3), possibly due to their extreme base composition.

### Discovering uncategorized bias

Finally, with the goal of understanding bias in the human genome that was not explained by our motifs, we generated >100-fold coverage of NA12878 using Illumina HiSeq data, from libraries generated with Kapa Biosystems reagents (Table 2, data set 14). We note that some apparently low or missing coverage will be due to true biological differences, including sequences that are present in the reference but not in NA12878. However, we used other deeply sequenced data sets and an assembly-based analysis to filter out many of these variant loci, as described below.

Initially we identified 5.5 Mb of the human reference sequence (HG19) having 0.1 or less relative coverage. If the data were unbiased, then 0.1 relative coverage would be more than 9 standard deviations from the expected coverage at each base. Therefore, we would expect no bases in the human genome to have such low coverage in the absence of sequencing bias. We then applied two filters to this 'undercovered set' to remove sequence that is unlikely to be present in the NA12878 genome (see Materials and methods). These filters, one based on analysis of the NA12878 assembly and the other based on a comparisons between NA12878 and a diverse population of other samples, excluded 8.7% (23 Mb) of the autosomal reference from further consideration. After this filtering, 3.6 Mb of undercovered reference genome remained.

Finally, because we were interested in discovering new bias contexts, we excluded regions that were similar (but not necessarily identical) to previously known motifs. Similarity was defined by matching at least one of the following motifs:

• GC ≤ 13%, 200-base regions in which the middle 100 bases have ≤13% GC content (a superset of the GC ≤ 10% motif);

• GC ≥ 70%, 200-base regions in which the middle 100 bases have ≥70% GC content (a superset of the GC ≥ 75% and GC ≥ 85% motifs);

• (AT)^10^, 130-base regions in which the middle 20 bases are repeated AT dinucleotides (a superset of the (AT)^15 ^motif);

• G|C ≥ 75%, 130-base regions in which the middle 30 bases are either 75% Gs or 75% Cs (a superset of the G|C ≥ 80% motif);

• the list of 1,000 'bad promoters'.

Except for the bad promoters, which were unaltered, these generalized motifs were selected to each cover roughly twice as many bases as their equivalents in the original motif list. Together they covered 2.8% (74 Mb) of the autosomal bases in HG19. The generalized motifs included 7.5% (1.7 Mb) of the bases previously excluded as probable biological variations. This enrichment may indicate that the biological variation filters excluded bases whose low coverage had a non-biological origin, or it may indicate a correlation between bias motifs and sites with high mutation rates.

Filtering out the probable biological differences between the sample and the reference and the areas similar to known motifs excluded 78% of the ten-fold undercovered locations in HG19. The remaining 35,389 undercovered intervals represented 0.045% (1.2 Mb) of the human autosomal reference genome with an N50 interval size of 98 bp.

**Figure 6:**
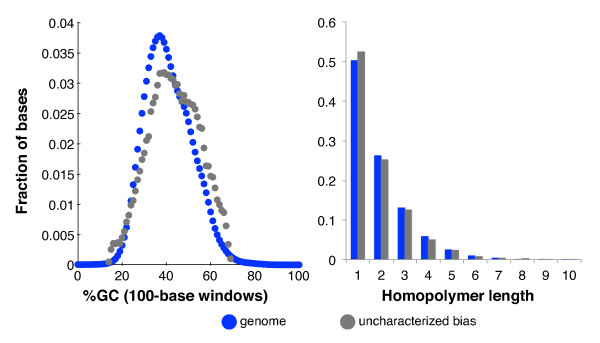
**GC and homopolymer distributions of uncharacterized Illumina undercoverage of human sample NA12878**. The graphs show the distribution of GC-content and homopolymer length for bases in the overall human genome and in the genome intervals that are ten-fold undercovered but which were not explained by known sequence biases or differences between the sample and reference sequence. Data are from Table 2, data set 14.

Performance on this fraction is hidden from our monitoring methods by its dissimilarity with the current set of motifs. On the Illumina HiSeq 'Kapa' data set, these bases had mean relative coverage of 0.037. They also suffered from high error rates - a mismatch rate of 0.020 (6.7 times the whole-genome average), a deletion rate of 0.11 (470 times the whole-genome average), and an insertion rate of 0.0021 (12 times the whole-genome average). The high deletion rate suggests that some of the undercoverage may have been due to short biological deletions in NA12878 relative to the reference sequence, but even if all the deletions originated in the sample, these regions would still be more than ten-fold undercovered. Their GC-content and homopolymer distributions did not differ appreciably from the overall genome (Figure 6). Clearly, these regions were either exceptionally resistant to the Illumina HiSeq technology or are places where the reference is inaccurate for NA12878 or for human samples generally. A list of the intervals' coordinates, GC content, and homopolymer N50 statistics are included in Additional file 6.

## Conclusions

Sequencing vendors and individual investigators alike strive to improve the quality of their data. This includes increasing read length, yield, overall base quality, and other average measures that reflect the behavior of the technology on 'typical' parts of the genome. However, such measures do not tell us how the technology performs on the 'hardest' parts of the genome, where data quality is lowest, and this is a critical omission. For example, as we have noted, in many human data sets there are large numbers of transcription start sites and first exons with essentially no coverage, and although this bias affects only a tiny fraction of the genome, it is of fundamental importance to the utility of the data.

A goal of our work has been to develop a systematic methodology for assaying coverage bias. We note the following key components of our approach.

Use of multiple microbial samples to assess bias: these samples span diverse sequence contexts and have finished reference sequences, thus facilitating analyses that expose 'extreme' regions on which performance is subpar. Their genomes are small and thus can be economically sequenced to high coverage.

Bias assessment on the human genome: because of its size and complexity, it spans even more diverse sequence contexts than the microbial samples. Conversely, although we used the highly studied sample NA12878, we note the lack of a truly finished sample-specific reference sequence that would facilitate definitive undercoverage analyses.

Formulation of a menagerie of 'bad' motifs: these encode known trouble spots, including high and low GC but also less well-known motifs, such as ATAT... runs. GC-bias plots effectively encode a whole family of motifs, one for each GC percentage. Motifs, especially on large genomes such as human, can be economically assayed using low-coverage data.

Use of relative coverage as the metric for coverage bias: whether assaying the whole genome or only motifs, relative coverage, as in Tables 2 and 3, simplifies and clarifies bias measurement.

We generated data from several technologies and applied this approach. We note the clear edge of single-molecule data from Pacific Biosciences, and that among the amplification-based technologies, data from Illumina had the lowest bias. However, our results represent a performance snapshot, and are exquisitely sensitive to the fine details of laboratory process (including library construction and sequencing), which we anticipate will continue to be improved. For example, we compared several methods of library construction and, within fixed protocols, noted assay variability arising from process fluctuation. We emphasize the ongoing importance of such process monitoring as a prerequisite for meaningful inferences about protocol improvements. Our experiences lead to the general conclusion that a platform's sequencing bias is not an immutable property. Although Ion Torrent's platform generally suffered from the most bias, reagent, protocol, and computational changes may lead to future improvements.

Bias in read accuracy (and not just coverage) is also important. It is well known that some loci on the genome sequence badly. Often this behavior is associated with polymerase slippage. In this work we defined assays, like those for coverage bias, which can be used to track error bias, and evaluated these assays on the same data sets used for coverage bias.

We note that bias is far from the only important metric for sequencing platform evaluation. Users must also evaluate accuracy, throughput, cost, speed, and many other factors when choosing the appropriate platform for an experiment. Indeed, there is no universal 'best' platform for every purpose.

Taken together, coverage and error bias assays provide a comprehensive view of bias in sequence data. We note several avenues for future work. First, the assays can be used to drive laboratory improvements, with the goal of minimizing bias. Second, the assays can be used to monitor intentional and unintentional process changes that might affect bias. Third, the assay genomes and our knowledge of them might be improved. In particular, it would be of great value to have an ultra-high-quality reference sequence for an available human sample. Fourth, the motifs might be refined and added to with the goal of creating as comprehensive and informative a list of bias-prone contexts as possible. Collectively these advances could improve data quality, thus increasing the accuracy and contiguity of genome assemblies and minimizing the likelihood that biologically important loci will be poorly represented in sequence data.

## Materials and methods

### Samples and references

*P. falciparum *3D7 DNA was provided by Daniel Neafsey (Broad Institute) and Sarah Volkman Cooke (Harvard School of Public Health). *E. coli *K12 and *R. sphaeroides *2.4.1 were provided by Louise Williams (Broad Institute). Human DNA samples (listed in the SAMPLE_ALIAS columns of the relevant spreadsheets in Additional file 7) were obtained from the NIGMS Human Genetic Repository and the NGHRI Sample Repository for Human Genetic Research collections at the Coriell Institute for Medical Research.

The references used for alignment were *E. coli *K12 substr. MG1655 (GenBank NC_000913.2), *R. sphaeroides *2.4.1 with plasmids (GenBank AKVW01000000), *P. falciparum *3D7 (GenBank GCA_000002765.1), and Human assembly 19/GRCh37 (GenBank GCA_000001405.1). The NA12878 diploid reference used to generate Table S2 in Additional file 3, Figure S1 in Additional file 4, and Figure S2 in Additional file 5 was created by the Gerstein Lab [37].

### Data

The SRA accession numbers for all the Illumina, Ion Torrent, and Pacific Biosciences data used in this work (data sets 1 to 15 and A1 to A11) are provided in Additional file 7. Each spreadsheet in the file corresponds to a data set referenced herein. The Complete Genomics data are publicly available [41].

### Illumina HiSeq and MiSeq sequencing

#### Illumina library construction

Illumina libraries indicated as 'low-input Fisher *et al*.' were prepared following the protocol described by Fisher *et al. *[31] with the following modifications: genomic DNA input into shearing was reduced from 3 µg to 100 ng in 50 µl volume. In addition, for adapter ligation, Illumina paired-end adapters were replaced with palindromic forked adapters with unique 8-base index sequences embedded within the adapter.

Libraries described as 'low-input Fisher *et al*. modified with Kapa Biosystems reagents' were made as described above except library construction and PCR reagents were obtained from Kapa Biosystems. DNA fragment end repair, A-base addition, and adapter ligation reactions were performed according to the manufacturer's recommendations (Kapa Biosystems, MA, catalog number KK8201) but utilizing the 'with-bead' SPRI-based clean up method in Fisher *et al*. Library enrichment with Kapa HiFi enzyme (catalog number KK2102) was performed as follows: the entire unenriched product was enriched in a reaction volume of 60 µl in the presence of 1× Kapa HiFi HF buffer, 0.4 mM each dNTP, 0.8 µM of each enrichment primer, and 1 unit of Kapa HiFi enzyme. Kapa HiFi PCR enrichment was performed for 8 cycles with the following cycling parameters: 98°C for 45 seconds; 8 cycles of 98°C for 15 seconds, 60°C for 30 seconds, 72°C for 30 seconds; 72°C for 1 minute.

Libraries described as 'Aird *et al*. with Phusion', 'Aird *et al*. with Phusion + betaine', and 'Aird *et al*. with AccuPrime', were generated as previously described [20].

Libraries described as 'Broad PCR-free' were prepared utilizing the protocol described as 'low-input Fisher *et al*. modified with Kapa Biosystems reagents' but with several modifications. The PCR-free protocol eliminates all of the amplification steps of the Fisher *et al*. protocol. Genomic DNA input into shearing was increased from 100 ng to 500 ng in 50 µl volume. Samples were sheared to an average fragment size of 200 bp instead of 150 bp. For adapter ligation, Illumina TruSeq Adapters (Illumina, CA, catalog FC-121-2001) were used instead of those described in Fisher *et al*.

For all Illumina PCR-based libraries prepared, the desired insert size was selected by gel electrophoresis with a target of ±10 to 15%. Multiple gel cuts were taken for libraries requiring high sequencing coverage. For the Broad PCR-free method, a second 0.7× SPRI reaction following adapter ligation was utilized instead of gel electrophoresis to tighten up size distribution and reduce excess adapter.

#### Illumina sequencing

Sequencing libraries were quantified using quantitative PCR (Kapa Biosystems, Woburn, MA, USA), normalized to 2 nM and denatured using 0.1 N NaOH prior to sequencing. Flowcell cluster amplification and sequencing were performed according to the manufacturer's protocols (Illumina, CA, USA) using HiSeq 2000 v2 (data sets 10 to 12, and A2), HiSeq 2000 v3 (data sets 13, 14, A1, and A3), HiSeq 2500 v1 (data sets A10 and A11), MiSeq v1 (data sets 1, 4, and 7), or Miseq v2 (data sets A4 to A9) cluster chemistry and flowcells. HiSeq data were analyzed using Illumina RTA v1.10.15 or RTA v.1.12.4.2. MiSeq data were analyzed using RTA v1.13 or v1.14.23. Read lengths were 2 × 251 bases for MiSeq data sets 1, 4, and 7; 2 × 150 bases for MiSeq data sets A4 to A9; 2 × 101 bases for HiSeq data sets 10 to 14 and A1 to A3; and 2 × 250 bases for HiSeq 2500 data sets A10 to A11. Data were further processed using the Picard data-processing pipeline [42] to generate BAM files. Alignment was performed using BWA version 0.5.9 [43]. The 'aln' command was run with the alignment options '-q 5 -l 32 -k 2 -o 1', followed by the 'sampe' command to generate a paired-end alignment. The Picard MarkDuplicates program was applied after alignment and all duplicate-flagged reads were excluded from the analyses in this manuscript. All human data sets, with the exception of data set 10 to 12, were also processed with the GATK IndelRealigner and TableRecalibration tools [44,45], but none of the results presented in this work depend on precise indel placement or on quality scores.

### Ion torrent sequencing

Libraries for Ion Torrent sequencing were created using the Ion Xpress™ Plus Fragment Library Kit, according to the Ion Xpress™ Plus gDNA Fragment Library Preparation protocol (version 5, Ion Torrent, Guilford, CT, USA).

Workflow parameters consisted of 100 ng DNA starting input material each of *P. falciparum*, *E. coli*, *R. sphaeroides*, and human, prepared independently in tubes. High molecular weight DNA was acoustically sheared to a size range of 100 to 1,000 bp using the following parameters: temperature, 6 to 8°C; duty cycle, 20% for *P. falciparum *and human, 1% for *E. coli *and *R. sphaeroides*; intensity, 5; cycles per burst, 200; time, 130 seconds for *P. falciparum *and human, 550 seconds for *E. coli *and *R. sphaeroides*; shearing tubes, MicroTubes crimpcap (Covaris, Woburn, MA, USA), using a Covaris E210 instrument. Size selection of the unamplified libraries was done with the Pippin Prep™ Instrument (SAGE Science, Beverly, MA, USA). The libraries were amplified following the protocol specifications for samples starting with 100 ng input. Final libraries were quantified and checked for size on an Agilent Bioanalyzer using the High Sensitivity DNA Kit (Agilent Technologies, Santa Clara, CA, USA).

Template preparation was conducted using the Ion PGM™ 200 Xpress™ Template Kit, following the Ion PGM™ 200 Xpress™ Template Kit protocol (version 3; Ion Torrent). Recovery of the Ion Spheres (ISPs) was done according to the Ion Sphere Particles 200 recovery protocol. Quality of the templated ISPs was assessed using the Guava easyCyte HT8 Cytometer (EMD Millipore, Billerica, MA, USA).

Sequencing of the samples was conducted according to the Ion PGM™ 200 Sequencing Kit Protocol (version 6; Ion Torrent). One or more 318 sequencing chips were loaded and run on an Ion Torrent PGM (Ion Torrent) for each sample. Each run was programmed to include 520 nucleotide flows to deliver 200-base reads, on average. Base calling and alignment were performed using the Torrent Suite 3.0 software (Ion Torrent).

Because the default TMAP aligner [46] cannot align to references with more than 4.3 billion bases, it was necessary to use the BWA-SW aligner [47] to realign the Ion data to the diploid NA12878 reference for the error-rate comparison presented in Table S2 in Additional file 3, Figure S1 in Additional file 4, and Figure S2 in Additional file 5. To ensure that the comparison was only affected by the choice of reference, we also used BWA-SW to realign the Ion data to Human assembly 19 (GRCh 37) for those comparisons. The alignment was done using version 0.6.2 of the aligner with default parameters and the '-M' option to generate only one primary alignment per read (the analysis ignores secondary alignments).

### Pacific Biosciences sequencing

Pacific Biosciences sequencing libraries were generated following the manufacturer's recommendations using the DNA Template Prep Kit Version 1 chemistry (Pacific Biosciences, Menlo Park, CA, USA) with the following modifications. For each sample, between 7 and 12 µg of genomic DNA was sheared to approximately 2 kb in size using a Covaris S instrument with the following parameters: temperature, 6 to 8°C; duty cycle, 20%; intensity, 0.1; cycles per burst, 1,000; time, 15 cycles × 60 seconds; shearing tubes, MiniTUBE-Clear (Covaris). DNA fragments were purified, end-repaired, and ligated with SMRTbell sequencing adapters following the manufacturer's recommendations (Pacific Biosciences) with the exception that the individual AMPure clean-up steps were purified three times rather than the recommended two. SMRTbell sequencing libraries were combined with sequencing primer and polymerase following the manufacturer's recommendations (Pacific Biosciences). The resulting complex was subjected to Pacific Biosciences sequencing, followed by primary data analysis (version 1.1.1 chemistry and analysis software) on the Pacific Biosciences RS instrument following the manufacturer's recommendations. Secondary analyses, including read filtering, were performed by SMRT Analysis versions 1.3.1 (*E. coli *and *P. falciparum*) or 1.3.0 (*R. sphaeroides*). Because Pacific Bioscience's BLASR aligner does not currently support random placement of ambiguously aligned reads, alignment was performed using the BWA-SW long-read aligner [47] version 0.6.2 with parameters '-b5 -q2 -r1 -z20 -M -w200'. BWA-SW parameters were based on the software's suggested defaults for Pacific Biosciences reads, adding the '-z20' parameter for greater accuracy (validated in [48]), the '-M' parameter to generate only one primary alignment per read (the analysis ignores secondary alignments), and '-w200' to encourage the aligner to generate only one alignment per read. The aligner input files were the 'filtered_subreads.fastq' files produced by the standard resequencing protocol.

### Complete Genomics data

All statistics were computed on BAM files provided by Complete Genomics. Complete Genomics' pipeline [49] first maps all reads that can be aligned to the reference with very few errors and then uses local assembly, constrained by read-pairing information, to accumulate evidence of variation from the remaining reads. Unlike the standard Complete Genomics BAM representations, these BAM files represent both the aligned and locally assembled reads, containing a single record for every read representing its highest-scoring alignment to the reference, using padded alignment to represent the relationships produced by the local assembler (personal communication, Srinka Ghosh, Complete Genomics). In cases where multiple equally good alignments/assemblies existed for a particular read pair, the file contains one chosen at random, similar to the policies of the aligners used on the other technologies. For the purpose of measuring coverage, this representation is superior to the BAMs produced by Complete Genomics' publicly available tools because it unifies the alignment and assembly data and presents a single 'best' alignment/assembly for each read pair.

### Selecting genomic regions

For microbial organisms, reads were aligned to the complete reference sequences, but only chromosomal contigs were considered in the bias calculations. For human data, reads were aligned to the complete reference sequence, but only autosomal contigs were considered in bias calculations. Plasmid, mitochondrial, and sex chromosomes were not included because they are not expected to be equimolar with the rest of the genome. Regions of the references containing ambiguous bases were also excluded from all bias computations as there is no way to accurately map reads to them or to assess their membership in motifs.

### Defining the bad promoters

The list of 'bad promoters' was identified based on data from 39 individuals sequenced on Illumina HiSeq v2 for the 1000 Genomes Project (198-fold total coverage, data set A2). To obtain the list, for each transcription-start site in the RefSeq database [50], the ratio of average coverage in the surrounding 200 bases to average coverage in the surrounding 3,000 bases was computed. Then the 1,000 sites with the lowest ratios were designated as 'bad promoters' and are listed in Additional file 1. If the database contained multiple entries for the same gene, the entry with the lowest coverage ratio was kept for the list. For comparison purposes, we used the same algorithm on a HiSeq v3 1000 Genomes data set (A3, 253-fold coverage of 71 individuals) to generate Additional file 2.

### Computing coverage and counting errors from alignments

All alignments were represented in the common SAM format (or its compressed, binary representation, BAM) [51]. In the SAM specification, the mapping between a read and a reference location is described by what is termed a 'CIGAR' string of operators. A read base that is aligned to a particular reference base is affiliated with the M, =, or × operators, as only those bases contribute to 'coverage' at a genome location.

For purposes of tracking error biases, we determined the number of CIGAR M, =, or X-mapped read bases where the read nucleotide differed from the reference nucleotide, and counted these as mismatches at the reference position. Similarly, deletions at a reference base were counted by incrementing a counter every time the CIGAR D operator is used to skip that base. Insertion errors are more problematic because these bases exist in the read but have no reference position. Some convention is necessary, so if an alignment contained an insertion of length L, denoted by 'LI' in the CIGAR string, we charged L insertions to the reference base immediately after the inserted sequence. For consistency, all error rates reported in the paper are computed relative to coverage levels: that is, error rates are fractions in which the numerator is the error count in a region or motif and the denominator is the number of mapped bases.

It is important to note that some details of the results may be sensitive to aligner algorithms and parameters. For example, a read whose best alignment has many errors may be left unmapped with one set of parameters, potentially contributing to coverage bias, or mapped with alternative parameters, potentially contributing to error bias. Similarly, parameters often determine whether a particular base is categorized as a mismatch, insertion, or deletion. As much as possible, we have addressed these issues by using aligners and parameters for each technology that have been validated as producing useful alignments for other applications. In our experience, the statistics we have presented are robust to reasonable substitutions of aligners and parameters.

### Filtering NA12878 data for the discovery of uncharacterized bias

The following filters were used to exclude regions for which low coverage of sample NA12878 were likely due to biological variation between the sample and the human reference.

We took the previously published NA12878 assembly, produced from a different set of Illumina data [52], and aligned its contigs to the HG19 reference. For each instance in which a contig in the NA12878 assembly contained a gap relative to the reference, we excluded the gap sequence from the undercovered set. Contigs from the ALLPATHS-LG assembly of NA12878 were aligned to the human reference hg19 with BWA-SW version 0.5.9 [38] using default arguments. Contigs longer than 100 kb were split before alignment so as to stay within the aligner's maximum read length. The splitting algorithm ensures that the resulting subsequences are no shorter than 50 kb. When BWA-SW detects large deletions in the contig-reads relative to the reference, it splits the alignments, treating the contigs as chimeric reads. Additionally, we scanned all the aligned contigs and marked any sliding 100-base windows that exhibited more than five alignment errors (mismatches, deleted bases, or inserted bases) as areas that may have high local rates of polymorphism. These regions are excluded from consideration because reads that cover them may fail to align to the reference, which would reduce apparent coverage even in the absence of sequencing bias.

The assembly-based analysis is limited to detection of variations that occur within contigs. To test for biological variations that might lie in assembly gaps, we identified genome locations that were well covered in data sets that mixed reads from diverse individuals, but were undercovered in multiple NA12878 data sets. First, we gathered two diverse sets of Illumina HiSeq sequencing data aligned to HG19: the first from 39 individuals (198-fold total, data set A2) from the 1000 Genomes Project [32] sequenced with version 2 chemistry and the second from 71 individuals (253-fold total, data set A3) from the 1000 Genomes Project sequenced with version 3 chemistry (see Data for SRA accession numbers). Any reference base with relative coverage of at least 0.5 in either diverse data set was considered 'well covered'. Second, we gathered three NA12878 Illumina HiSeq data sets aligned to HG19: 152-fold from HiSeq v2 chemistry (the Phusion, Phusion + betaine, and AccuPrime data discussed previously, data sets 10 to 12), 110-fold from version 3 chemistry using low-input Fisher *et al*. library construction (data set 13 with four additional lanes from data set A1), and 120-fold from version 3 chemistry with Kapa-based library construction (the previously discussed 'Kapa' data set 14). Any reference base with less than 0.1 relative coverage in all three NA12878 data sets was considered 'undercovered'. This was a subset of the bases that are undercovered in the HiSeq 'Kapa' data: if a base was not undercovered in one of the other two data sets, then we assumed that its bad performance in the 'Kapa' data might be due to technology rather than biology.

Any genome base that was well covered in the diverse data and undercovered in all of the NA12878 data was then removed from further analysis as a potential biological variation.

## Abbreviations

bp: base pair; NCS1: neuronal calcium sensor-1; PCR: polymerase chain reaction; SNP: single-nucleotide polymorphism.

## Competing interests

The authors declare that they have no competing interests.

## Authors' contributions

MGR performed all the analyses and wrote the paper. DBJ conceived of many of the analyses: directed the project: and extensively revised the paper. CR and CN contributed analytic ideas and helped craft and edit the manuscript. MGR: CR: CN and DBJ jointly devised the experimental design. MC: AH: NJL: and RH wrote and edited portions of the text: designed and developed lab protocols: and provided intellectual input. All authors read and approved the final manuscript.

## Supplementary Material

Additional file 1 **The 'bad promoters' list for Human assembly 19 (GRCh 37), as described in the main text and method section, computed from HiSeq v2 data set A2; intervals are annotated with gene names and the coverage ratios used to select them (see Materials and methods for details)**. Click here for file

Additional file 2 **The 'bad promoters' list for Human assembly 19 (GRCh 37), as described in the main text and method section, computed from HiSeq v3 data set A3; intervals are annotated with gene names and the coverage ratios used to select them (see Materials and methods for details)**. Click here for file

Additional file 3 **The supplementary tables referred to in the text**. Click here for file

Additional file 4 Figure S1 - Human error rates as a function of GC composition and reference. Each graph shows mismatch (light blue), deletion (dark blue), and insertion (maroon) rates (y-axis) as a function of GC composition (x-axis). Data are shown for the human NA12878 sample sequenced by Illumina HiSeq (Table 2, data set 14) and Ion Torrent PGM (Table 2, data set 15) aligned both to the standard Human assembly 19 (GRCh37) reference and to the NA12878-specific diploid reference created by the Gerstein lab [37]. Error rates are only plotted for GC percentages for which there are at least 1,000 100-base windows in Human assembly 19. Click here for file

Additional file 5 Figure S2 - Human error rates as a function of homopolymer length and reference. Each graph shows mismatch (light blue), deletion (dark blue), and insertion (maroon) rates (y-axis) within homopolymers of various lengths (x-axis). Data are plotted from human sample NA12878 as sequenced by Illumina HiSeq (Table 2, data set 14) and Ion Torrent PGM (Table 2, data set 15) and aligned both to the standard Human assembly 19 (GRCh37) reference and to the NA12878-specific diploid reference created by the Gerstein lab [37]. Click here for file

Additional file 6 **The intervals of the human reference that had less than 0**.1 relative coverage in data set 14 and could not be categorized as biological variations or as similar to known bias motifs. Also included are the GC content fraction and homopolymer N50 for each interval. Click here for file

Additional file 7 **The SRA numbers for all Illumina, Ion Torrent, and Pacific Biosciences data used in the paper**. Click here for file
