# Supplementary material for: Characterizing and measuring bias in sequence data
Source: Genome Biol. 2013 May 29;14(5):R51. doi: 10.1186/gb-2013-14-5-r51 (PMC4053816; doi:10.1186/gb-2013-14-5-r51)
Supplement: Additional file 3 — The supplementary tables referred to in the text. [file gb-2013-14-5-r51-S3.DOCX]

## Table S1 – Coverage bias comparison of Fisher et al. and PCR-free library construction

| **Data set** | | | | | **Relative coverage** | | | | | |
| --- | --- | --- | --- | --- | --- | --- | --- | --- | --- | --- |
|  |  |  |  |  | **GC extremes** | | | **Special motifs** | | |
| **Sample** | **#** | **Library method** | **Sequencing platform** | **Coverage (x)** | **GC ≤ 10%** | **GC ≥ 75%** | **GC ≥ 85%** | **(AT)^15^** | **G\|C ≥ 80%** | **Bad promoters** |
| *P. falciparum*  3D7 | A4 | Fisher et al.* with Kapa reagents | Illumina MiSeq | 21 | 0.77 | — | — | 0.61 | — | — |
|  | A5 | Broad PCR-free |  | 12 | 0.93 | — | — | 0.88 | — | — |
| *E. coli*  K12 MG1655 | A6 | Fisher et al.* with Kapa reagents |  | 21 | — | 0.76 | — | — | — | — |
|  | A7 | Broad PCR-free |  | 08.9 | — | 0.94 | — | — | — | — |
| *R. sphaeroides*  2.4.1 | A8 | Fisher et al.* with Kapa reagents |  | 12 | — | 0.83 | 0.23 | — | — | — |
|  | A9 | Broad PCR-free |  | 08.1 | — | 0.94 | 0.77 | — | — | — |
| Human  NA12878 | A10 | Fisher et al.* with Kapa reagents | Illumina HiSeq 2500 | 30 | 0.52 | 0.89 | 0.59 | 0.38 | 0.60 | 0.44 |
|  | A11 | Broad PCR-free |  | 47 | 0.85 | 0.71 | 0.65 | 0.63 | 0.44 | 0.53 |

*low-input variation of Fisher et al. (see Methods)

Data sets comparing Fisher et al. and Broad PCR-free library preparation protocols, along with their total coverage of the genome, and relative coverage, for each of five bias motifs and a set of ‘bad promoters’ (see text). Entries are blank if the samples’ genome had no instances of the given motif.

## Table S2 – Comparison of human sequencing error rate using a sample-specific reference

| **Sample** | **#** | **Sequencing platform** | **Reference** | **Mismatches** | **Deletions** | **Insertions** | **Total** |
| --- | --- | --- | --- | --- | --- | --- | --- |
| Human NA12878 | 14 | Illumina HiSeq v3 | Human assembly 19 | 0.0030 | 0.00023 | 0.00017 | 0.0031 |
|  | 14 |  | Gerstein diploid NA12878 | 0.0018 | 0.000052 | 0.000027 | 0.0019 |
|  | 15 | Ion Torrent PGM | Human assembly 19 | 0.0048 | 0.0063 | 0.0050 | 0.016 |
|  | 15 |  | Gerstein diploid NA12878 | 0.0048 | 0.0062 | 0.0049 | 0.016 |

The error rates computed for Illumina HiSeq and Ion Torrent PGM sequencing of human sample NA12878 aligned to the standard human reference (Human assembly 19 / GRCh37) and aligned to a diploid NA12878-specific reference (Gerstein Lab, available at <http://sv.gersteinlab.org/NA12878_diploid/NA12878_diploid_dec16.2012.zip>). Note that the Ion Torrent data were aligned to both references using BWA-SW, rather than the TMAP aligner used in the main text of the paper (see Methods).

## Table S3 – Error rate comparison of Fisher et al. and PCR-free library construction

| **Sample** | **#** | **Library method** | **Mismatches** | **Deletions** | **Insertions** | **Total** |
| --- | --- | --- | --- | --- | --- | --- |
| *P. falciparum* 3D7 | A4 | Fisher et al.* with Kapa reagents | 0.0026 | 0.00028 | 0.00015 | 0.0031 |
|  | A5 | Broad PCR-free | 0.0025 | 0.00013 | 0.000057 | 0.0027 |
| *E. coli* K12 MG1655 | A6 | Fisher et al.* with Kapa reagents | 0.0022 | 0.0000095 | 0.0000048 | 0.0022 |
|  | A7 | Broad PCR-free | 0.0023 | 0.000012 | 0.0000051 | 0.0023 |
| *R. sphaeroides* 2.4.1 | A8 | Fisher et al.* with Kapa reagents | 0.0030 | 0.000013 | 0.000010 | 0.0030 |
|  | A9 | Broad PCR-free | 0.0028 | 0.000016 | 0.0000089 | 0.0028 |
| Human NA12878 | A10 | Fisher et al.* with Kapa reagents | 0.0027 | 0.00027 | 0.00020 | 0.0032 |
|  | A11 | Broad PCR-free | 0.0043 | 0.00023 | 0.00018 | 0.0047 |

*low-input variation of Fisher et al. (see Methods)

The error rates of microbial and human libraries created with the low-input Fisher et al. library construction protocol (with Kapa reagents) and the Broad PCR-free protocol. The microbial libraries (data sets #A4-A9) were sequenced on the same MiSeq flowcell, the human libraries (data sets #A10 and #A11) were sequenced on separate, but closely matched, HiSeq 2500 flowcells.
