# Supplementary figures and images for: Characterizing and measuring bias in sequence data
Source: Genome Biol. 2013 May 29;14(5):R51. doi: 10.1186/gb-2013-14-5-r51 (PMC4053816; doi:10.1186/gb-2013-14-5-r51)

Human assembly 19

NA12878 diploid

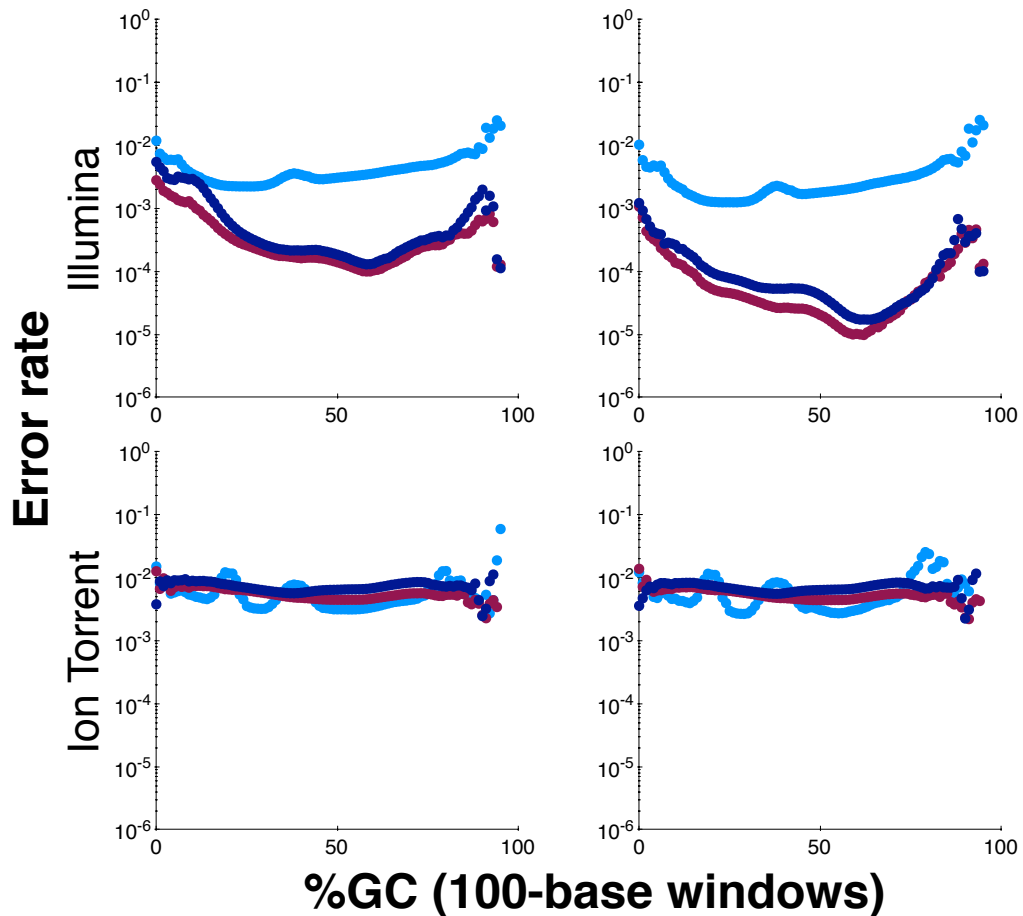

Supplement: Additional file 4 — Figure S1 - Human error rates as a function of GC composition and reference. Each graph shows mismatch (light blue), deletion (dark blue), and insertion (maroon) rates (y-axis) as a function of GC composition (x-axis). Data are shown for the human NA12878 sample sequenced by Illumina HiSeq (Table 2, data set 14) and Ion Torrent PGM (Table 2, data set 15) aligned both to the standard Human assembly 19 (GRCh37) reference and to the NA12878-specific diploid reference created by the Gerstein lab [37]. Error rates are only plotted for GC percentages for which there are at least 1,000 100-base windows in Human assembly 19. [file gb-2013-14-5-r51-S4.PDF]
