# Supplementary material for: Characterizing and measuring bias in sequence data
Source: Genome Biol. 2013 May 29;14(5):R51. doi: 10.1186/gb-2013-14-5-r51 (PMC4053816; doi:10.1186/gb-2013-14-5-r51)
Supplement: Additional file 5 — Figure S2 - Human error rates as a function of homopolymer length and reference. Each graph shows mismatch (light blue), deletion (dark blue), and insertion (maroon) rates (y-axis) within homopolymers of various lengths (x-axis). Data are plotted from human sample NA12878 as sequenced by Illumina HiSeq (Table 2, data set 14) and Ion Torrent PGM (Table 2, data set 15) and aligned both to the standard Human assembly 19 (GRCh37) reference and to the NA12878-specific diploid reference created by the Gerstein lab [37]. [file gb-2013-14-5-r51-S5.PDF]

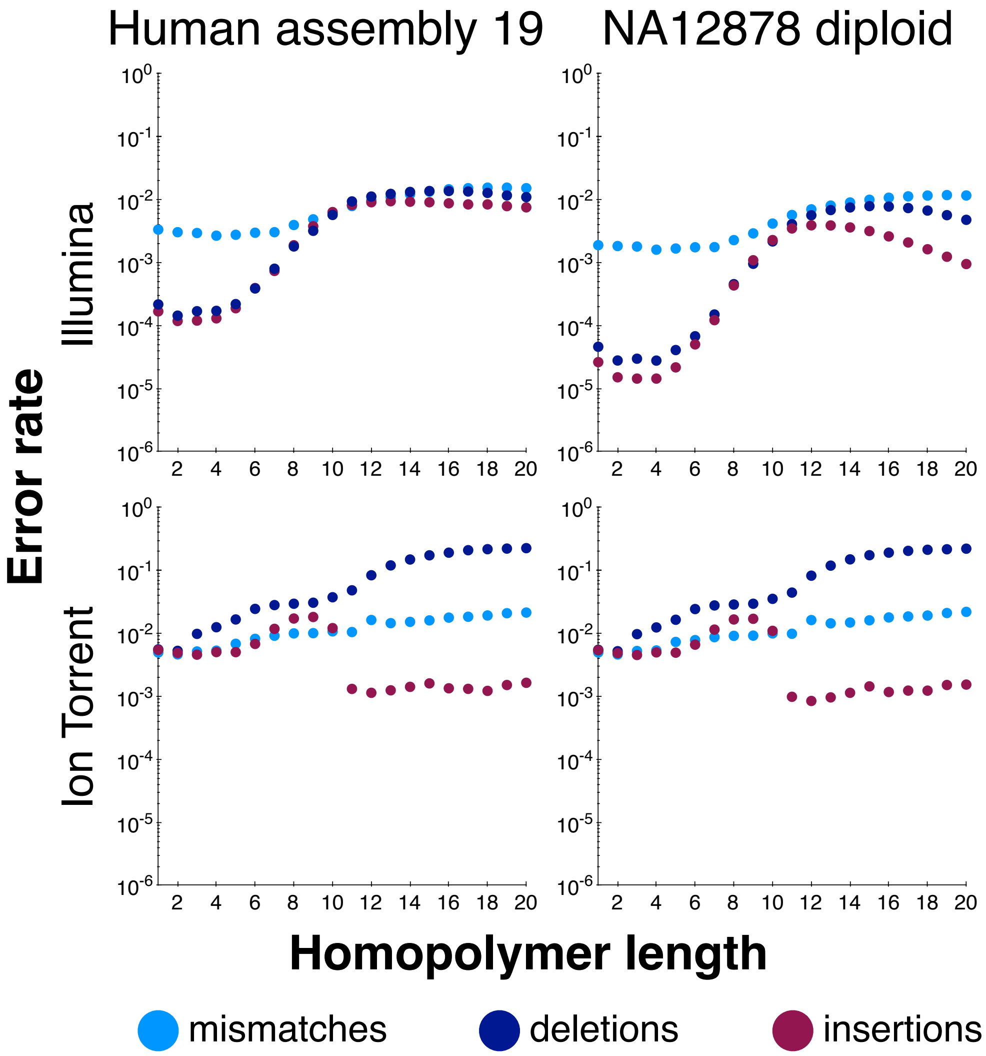

Human assembly 19
NA12878 diploid
Illumina
Ion Torrent
Error rate
Homopolymer length
10^0
10^{-1}
10^{-2}
10^{-3}
10^{-4}
10^{-5}
10^{-6}
2 4 6 8 10 12 14 16 18 20
mismatches
deletions
insertions
